# Supplementary material for: A Freely Available, Self-Calibrating Software for Automatic Measurement of Freezing Behavior
Source: Front Behav Neurosci. 2019 Sep 13;13:205. doi: 10.3389/fnbeh.2019.00205 (PMC6753174; doi:10.3389/fnbeh.2019.00205)
Supplement: Supplementary file 1 [file Table_1.DOCX]

| **Set 1** |  | **Adjusted P Value** |
| --- | --- | --- |
| MxA (Intra) vs. MxM |  | 0.1761 |
| MxA (Intra) vs. AxA |  | 0.5399 |
| MxA (Intra) vs. MxA (Inter) |  | 0.9753 |
| MxM vs. AxA |  | 0.7221 |
| MxM vs. MxA (Inter) |  | 0.0059 |
| AxA vs. MxA (Inter) |  | 0.0779 |
| **Set 2** |  |  |
| MxA (Intra) vs. MxM |  | <0.0001 |
| MxA (Intra) vs. AxA |  | <0.0001 |
| MxA (Intra) vs. MxA (Inter) |  | 0.9912 |
| MxM vs. AxA |  | 0.9985 |
| MxM vs. MxA (Inter) |  | <0.0001 |
| AxA vs. MxA (Inter) |  | <0.0001 |
| **Set 3** |  |  |
| MxA (Intra) vs. MxM |  | 0.0342 |
| MxA (Intra) vs. AxA |  | 0.6124 |
| MxA (Intra) vs. MxA (Inter) |  | 0.9996 |
| MxM vs. AxA |  | 0.254 |
| MxM vs. MxA (Inter) |  | 0.0061 |
| AxA vs. MxA (Inter) |  | 0.4656 |
| **Set 4** |  |  |
| MxA (Intra) vs. MxM |  | 0.0009 |
| MxA (Intra) vs. AxA |  | 0.178 |
| MxA (Intra) vs. MxA (Inter) |  | >0.9999 |
| MxM vs. AxA |  | 0.0668 |
| MxM vs. MxA (Inter) |  | <0.0001 |
| AxA vs. MxA (Inter) |  | 0.0509 |

**Table S1. Statistical comparisons between specific groups using manual or automated scoring.** To compare the distribution of *r* values. we analyzed the statistical comparison between groups using Tukey’s multiple comparisons test using α = 0.05.
