# Supplementary material for: A Freely Available, Self-Calibrating Software for Automatic Measurement of Freezing Behavior
Source: Front Behav Neurosci. 2019 Sep 13;13:205. doi: 10.3389/fnbeh.2019.00205 (PMC6753174; doi:10.3389/fnbeh.2019.00205)
Supplement: Supplementary file 2 [file Data_Sheet_1.PDF]

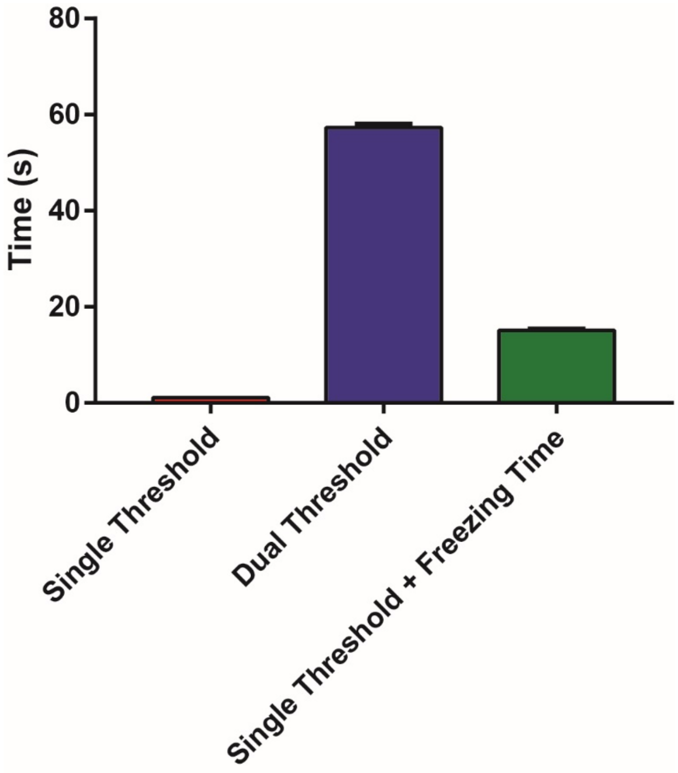

Figure S1. Processing time for calibration using different parameters. Comparison of mean time spent to calibrate the system (y axis) using different parameters: variable single threshold (red), variable dual threshold (blue) or variable single threshold and variable minimum freezing time. Bars represent the mean time (± SEM) for calibration using each video from the 4 video sets in an AMD Phenom II X4 965 Quadcore processor and 16gb RAM memory running Windows 10 (single threshold: 1.1 ± 0.02; dual threshold: 57.3 ± 0.7; single threshold + minimum freezing time: 15.1 ± 0.2, One-way ANOVA, $p < 0.0001$; single threshold vs dual threshold, $p < 0.0001$; single threshold vs single threshold + freezing time, $p < 0.0001$; dual threshold vs single threshold + freezing time, $p < 0.0001$, Tukey’s post-hoc; n = 54 2-min videos/group).
