# Supplementary material for: A Freely Available, Self-Calibrating Software for Automatic Measurement of Freezing Behavior
Source: Front Behav Neurosci. 2019 Sep 13;13:205. doi: 10.3389/fnbeh.2019.00205 (PMC6753174; doi:10.3389/fnbeh.2019.00205)
Supplement: Supplementary file 3 [file Data_Sheet_2.PDF]

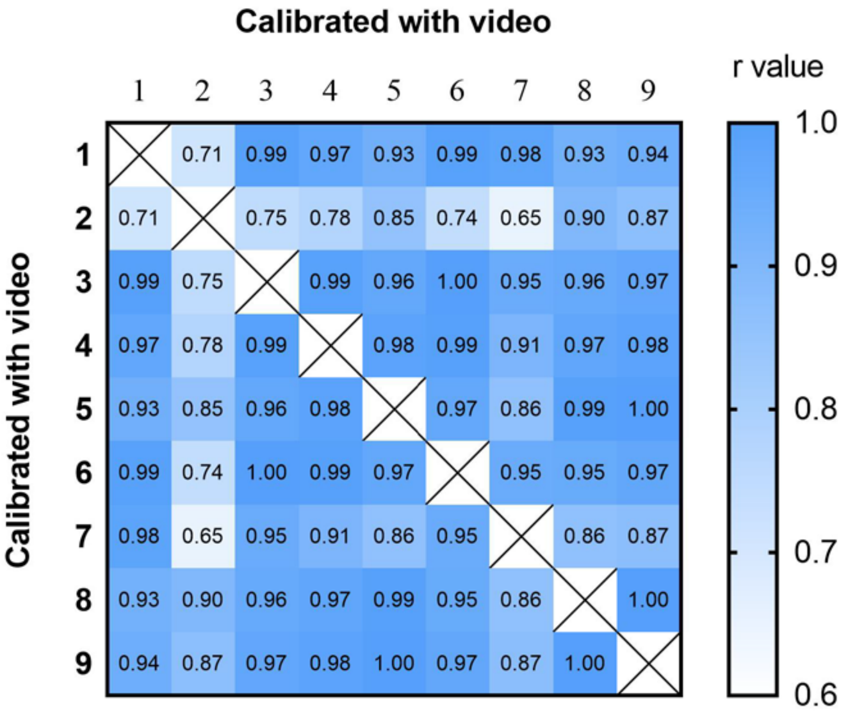

Figure S2. Intra-user variability in automated scoring according to calibration video. Heat maps show correlation matrix (*r* values) between automated assessments based on each video for set 1 for one observer. For this process, only videos that passed the minimum criteria were used to calibrate the system (n = 9). The largest *p* value obtained for the correlations was 0.02 (video 2 vs. video 7), while all other *p* values were below 0.006.
